# Supplementary figures and images for: Machine learning-based prediction of in-hospital mortality using admission laboratory data: A retrospective, single-site study using electronic health record data
Source: PLoS One. 2021 Feb 5;16(2):e0246640. doi: 10.1371/journal.pone.0246640 (PMC7864463; doi:10.1371/journal.pone.0246640)

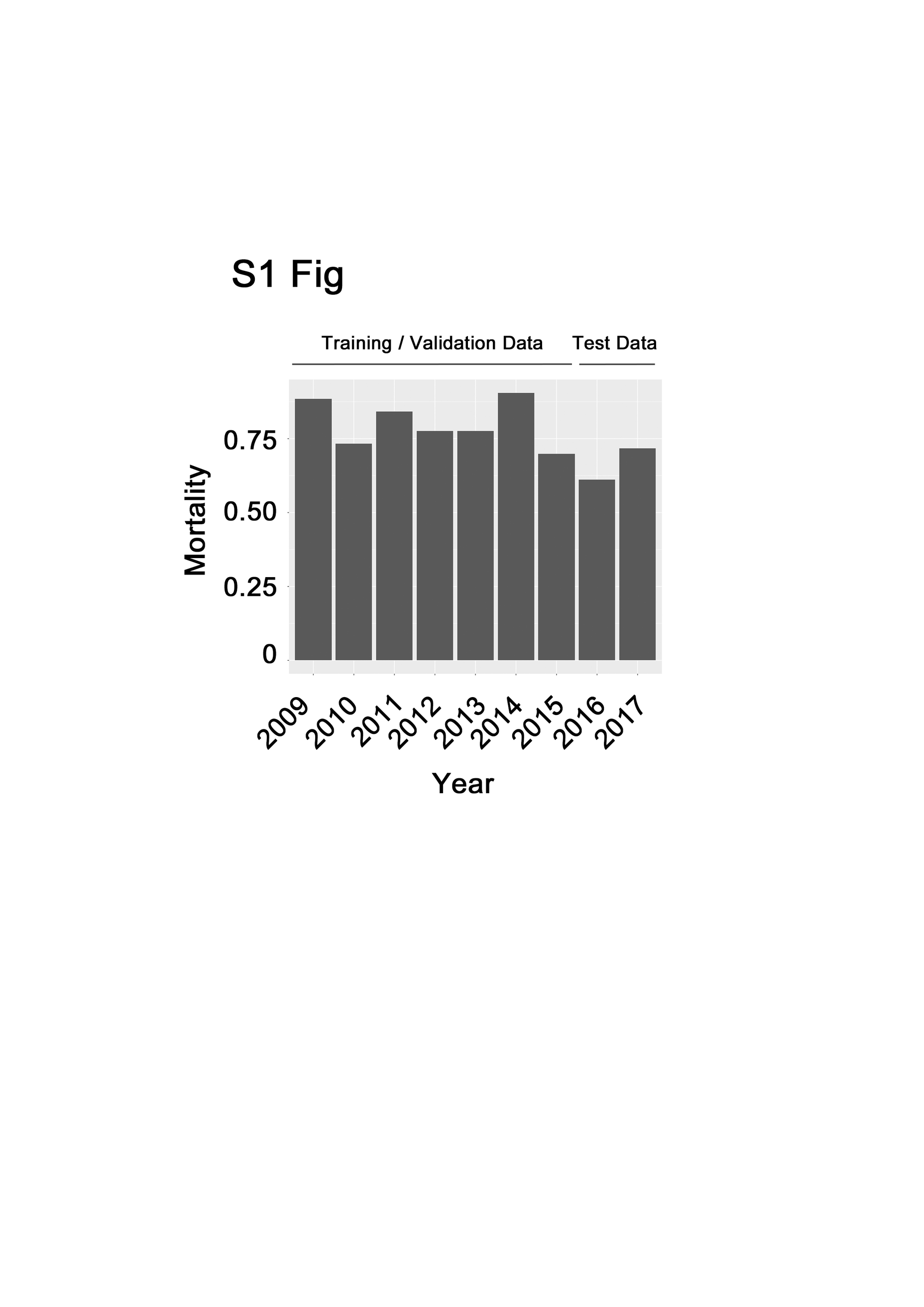

Supplement: S1 Fig — The bars show the in-hospital mortality rates for each year. (TIF) [file pone.0246640.s001.tif]

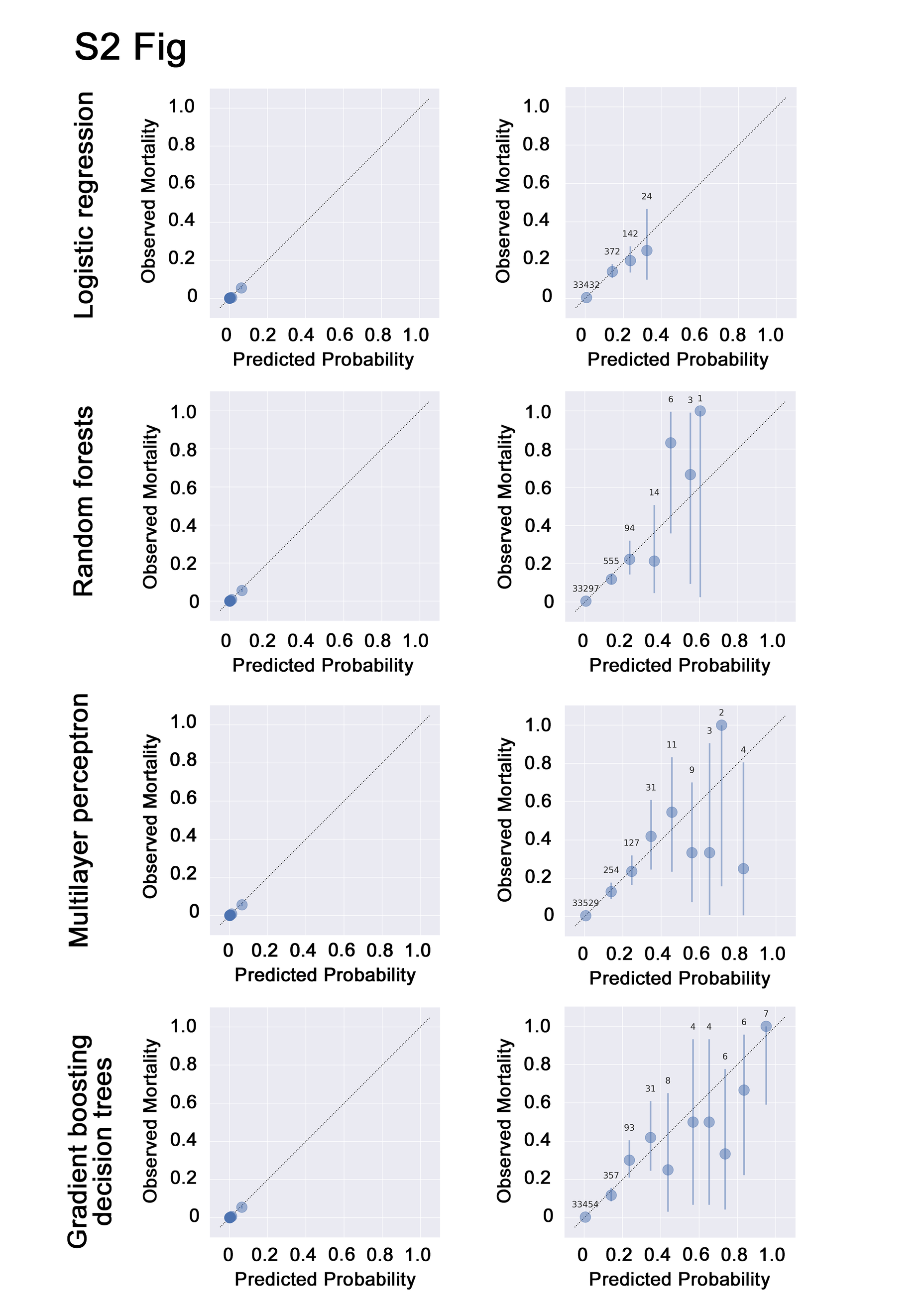

Supplement: S2 Fig — On the left side of the figure, each plot figure shows the observed probability and predicted probability based on 10 quantiles of predicted probability. On the right side of the figure, each plot figure shows the observed probability and predicted probability based on 10 equal parts of predicted probability. Confidence intervals are calculated using the F distribution. (TIF) [file pone.0246640.s002.tif]
