# Supplementary material for: Machine learning-based prediction of in-hospital mortality using admission laboratory data: A retrospective, single-site study using electronic health record data
Source: PLoS One. 2021 Feb 5;16(2):e0246640. doi: 10.1371/journal.pone.0246640 (PMC7864463; doi:10.1371/journal.pone.0246640)
Supplement: S1 Table — On the left side of the columns of each data, continuous variables are presented as the mean value and standard deviation (SD) after imputation, and categorical variables are shown as percentages after imputation. Missing rates before imputation are presented as percentages on the right side of the columns. (DOCX) [file pone.0246640.s003.docx]

S1 Table. Baseline Characteristics of variables

|  | **Training/validation data**  **(n=119,160)** | | **Test data**  **(n=33,970)** | |
| --- | --- | --- | --- | --- |
|  | Mean value (SD)  or  proportion (%) | Missing  (%) | Mean value (SD)  or  proportion (%) | Missing  (%) |
| Sex (Male %) | 51.85 | 0 | 51.44 | 0 |
| Age (year, mean (SD)) | 61.58 (16.42) | 0 | 61.29 (17.03) | 0 |
| Hospitalization (day, mean (SD)) | 14.70 (23.51) | 0 | 13.81 (17.14) | 0 |
| Whole blood cell count (x10^3^/µL, mean (SD)) | 6.82 (5.83) | 1.08 | 7.27 (6.49) | 0.96 |
| Red blood cell count (x10^4^/µL, mean (SD)) | 400.44 (71.49) | 1.08 | 409.57 (73.22) | 0.96 |
| Whole blood hemoglobin (g/dL, mean (SD)) | 12.14 (2.15) | 1.08 | 12.27 (2.15) | 0.96 |
| Hematocrit (%, mean (SD)) | 37.15 (6.07) | 1.08 | 37.70 (6.13) | 0.96 |
| Mean corpuscular volume (fL, mean (SD)) | 93.26 (6.69) | 1.08 | 92.58 (6.68) | 0.96 |
| Mean corpuscular hemoglobin (pg, mean (SD)) | 30.44 (2.62) | 1.08 | 30.11 (2.61) | 0.96 |
| Platelet count (x10^4^/µL, mean (SD)) | 22.55 (9.35) | 1.08 | 23.36 (9.57) | 0.96 |
| Serum albumin (g/dL, mean (SD)) | 3.76 (0.57) | 6.14 | 3.81 (0.56) | 4.19 |
| Serum total bilirubin (mg/dL, mean (SD)) | 0.85 (1.32) | 9.16 | 0.83 (1.30) | 7.75 |
| Serum aspartate aminotransferase (IU/L, mean (SD)) | 36.03 (94.84) | 3.54 | 35.93 (124.89) | 2.55 |
| Serum alanine aminotransferase (IU/L, mean (SD)) | 29.70 (78.37) | 2.92 | 29.23 (87.27) | 2.28 |
| Serum lactate dehydrogenase (IU/L, mean (SD)) | 251.69 (208.93) | 9.04 | 248.80 (193.32) | 7.31 |
| Alkaline phosphatase (U/L, mean (SD)) | 317.14 (310.19) | 18.89 | 306.32 (296.91) | 17.17 |
| Gamma-glutamyltransferase (IU/L, mean (SD)) | 68.24 (139.39) | 16.27 | 70.55 (146.68) | 16.42 |
| Serum sodium (mEq/L, mean (SD)) | 138.99 (3.46) | 5.42 | 139.18 (3.40) | 4.28 |
| Serum potassium (mEq/L, mean (SD)) | 4.23 (0.50) | 5.16 | 4.22 (0.51) | 4.08 |
| Serum chloride (mEq/L, mean (SD)) | 104.18 (3.82) | 11.64 | 103.25 (3.82) | 9.92 |
| Blood urea nitrogen (mg/dL, mean (SD)) | 18.39 (12.48) | 3.15 | 17.92 (12.16) | 2.59 |
| Serum creatinine (mg/dL, mean (SD)) | 1.00 (1.18) | 2.94 | 1.01 (1.21) | 2.32 |
| Estimated glomerular filtration rate (mL/min/1.73m^2^, mean (SD)) | 74.13 (35.20) | 2.95 | 73.82 (39.94) | 2.34 |
| Serum C-reactive protein (mg/dL, mean (SD)) | 1.55 (4.11) | 3.87 | 1.58 (4.18) | 3.14 |
| Leave hospital mortality within 14 days (mortality %) | 0.84 | 0 | 0.69 | 0 |
